# Epigenetic Regulation of Dental Pulp Stem Cell Fate

**DOI:** 10.1155/2020/8876265

**Published:** 2020-10-13

**Authors:** Dan Zhou, Lu Gan, Yiran Peng, Yachuan Zhou, Xin Zhou, Mian Wan, Yi Fan, Xin Xu, Xuedong Zhou, Liwei Zheng, Wei Du

**Affiliations:** ^1^State Key Laboratory of Oral Diseases, National Clinical Research Center for Oral Diseases, Department of Pediatric Dentistry, West China Hospital of Stomatology, Sichuan University, Chengdu, Sichuan 610041, China; ^2^State Key Laboratory of Oral Diseases, National Clinical Research Center for Oral Diseases, Department of Cariology and Endodontics, West China Hospital of Stomatology, Sichuan University, Chengdu, Sichuan 610041, China

## Abstract

Epigenetic regulation, mainly involving DNA methylation, histone modification, and noncoding RNAs, affects gene expression without modifying the primary DNA sequence and modulates cell fate. Mesenchymal stem cells derived from dental pulp, also called dental pulp stem cells (DPSCs), exhibit multipotent differentiation capacity and can promote various biological processes, including odontogenesis, osteogenesis, angiogenesis, myogenesis, and chondrogenesis. Over the past decades, increased attention has been attracted by the use of DPSCs in the field of regenerative medicine. According to a series of studies, epigenetic regulation is essential for DPSCs to differentiate into specialized cells. In this review, we summarize the mechanisms involved in the epigenetic regulation of the fate of DPSCs.

## 1. Introduction

Epigenetics, defined as “the study of changes in gene function that are mitotically and/or meiotically heritable and that do not entail a change in DNA sequence” [1], has gradually become a research hotspot in recent decades. Epigenetic regulation can influence gene expression without modifying the primary DNA sequence. Therefore, two cells, containing the same genetic information, can behave totally differently [2]. The principal epigenetic mechanisms, comprising DNA methylation, histone modifications, and those mediated by noncoding RNAs (ncRNAs), have been proved to perform an essential role in the differentiation, proliferation, and behavior of cells [3–5].

Stem cells (SCs) are a unique population of cells which provide progenitor cells via dividing and proliferating throughout postnatal life, which in turn differentiate into specialized cells in most tissues of the body [6]. Mesenchymal stem cells (MSCs), a heterogenic cell population, can be isolated from various tissues and are noted for their multipotency differentiation potential [7]. A group of MSCs, mainly including dental pulp stem cells (DPSCs), periodontal ligament stem cells (PDLSCs), dental follicle progenitor cells (DFPCs), stem cells from exfoliated deciduous teeth (SHED), and stem cells from the apical papilla (SCAP), are derived from dental tissues. Among them, DPSCs, which are originated from dental pulp of permanent teeth, play a critical role in restorative dentin formation and pulp homeostasis. Since DPSCs were first isolated in 2000 by Gronthos et al. [8], numerous studies have identified the self-renewal and multilineage differentiation ability of DPSCs, such as odontogenic, angiogenic, osteogenic, neurogenic, myogenic, adipogenic, and chondrogenic differentiation [9–11]. These unique characteristics make DPSCs applicable in regenerative medicine [12]. Hence, it is vital to investigate the factors that modulate the fate of DPSCs, including their proliferation and differentiation. According to a series of studies, epigenetic regulation is closely related to DPSC fate [11–20]. This literature reviews the general characteristics, immunophenotypes, and multipotential differentiation of DPSCs and current progress on the epigenetic regulation in the determination of DPSC fate.

## 2. Epigenetics

Epigenetics was first proposed to describe the complicated development process from genotype to phenotype by Conrad Waddington in 1942 [13]. Epigenetic regulation can alter the state of chromatin without changing the DNA sequence, thereby affecting access to genes within the cell [2]. When the environment around the cell changes, the initiator such as ncRNAs receives an epigenator signal and determines the location on the chromosome where the chromatin state needs to be changed, thereby affecting gene expression. The epigenetic maintainers, including DNA methylation and histone modifications, sustain the epigenetic state of chromatin and allow it to be inherited [14, 15].

### 2.1. DNA Methylation

DNA methylation is a stable and inheritable epigenetic mark that modulates the chromatin structure and gene expression. DNA methyltransferases (DNMTs), including DNMT1, DNMT3A, DNMT3B, and DNMT3L, are the enzymes responsible for DNA methylation. Among them, DNMT1 plays a crucial part in maintaining DNA methylation during the course of DNA replication, while DNMT3A and DNMT3B target unmethylated CpGs and are responsible for *de novo* DNA methylation [16–18]. Moreover, DNMT3L stimulates the DNA methylation activity of DNMT3A and DNMT3B [19–21]. DNA demethylation can be achieved by diluting methylation markers during DNA replication, or independently of DNA replication. The latter requires the involvement of ten-eleven translocation proteins (TET1, TET2, and TET3) and the activation-induced deaminase [22]. DNA methylation leads to gene silencing by arresting the binding of transcriptional factors or by chromatin remodeling, and its dynamics is involved in diverse biological processes [23–25]. Thus, DNA methylation plays a vital part in stem cell differentiation, development, and disease by regulating genes [26–28].

### 2.2. Histone Modifications

Histone proteins, including H1, H2A, H2B, H3, and H4, along with DNA form nucleosomes, which are referred to as the basic units of chromatin. Studies have confirmed that the lack of histones in the promoter region is essential for transcriptional activation [29]. The posttranscriptional modifications (PTMs) of the amino acids on the histone tails and cores, comprising methylation, acetylation, ubiquitination, phosphorylation, ADP-ribosylation, and glycation, are widely reported to be critical for the chromatin architecture, nucleosome stability, and transcription of genes [30–34].

Among these histone modifications, methylation and acetylation are the most widely studied. Histone methylation, catalyzed by histone methylases and demethylases (HDMs), can occur at multiple sites of histones, mainly on lysine and arginine residues [35]. Histone methylation activates or inhibits transcription depending on the location and methylation status. Similarly, histone acetylation is regulated by histone deacetylases (HDACs) and histone acetyltransferases (HATs) and is always related to active genes [36]. These different histone modifications have crosstalk with each other and constitute a regulatory network that regulates gene transcriptional activity by affecting the chromatin structure, thereby affecting development, diseases, and stem cell fate [30, 34, 37].

### 2.3. ncRNAs

ncRNAs consist of a group of RNAs that do not encode proteins. They include housekeeping ncRNAs and regulatory ncRNAs. Notably, the regulatory ncRNAs, composed of micro-RNAs (miRNAs), endogenous small interfering RNAs (siRNAs), PIWI interacting RNAs (piRNAs), and long noncoding RNAs (lncRNAs), are critical for epigenetic control [38]. According to their length, the ncRNAs are categorized as lncRNAs and short ncRNAs, which include miRNAs, piRNAs, and siRNAs. lncRNAs, with the length of more than 200 nucleotides (nt), can regulate gene expression through transvection, chromatin modification, and transcriptional and posttranscriptional regulation [39, 40]. miRNA, a sequence of single-stranded RNA about 22 nt in length, can degrade mRNA or repress translation to silence the gene through tie to the 3′-untranslated regions (3′-UTRs) of the particular mRNA [41]. Therefore, ncRNAs can epigenetically regulate gene expression at different levels.

### 2.4. Epigenetic Network

There are also some crosstalks between these epigenetic mechanisms that modulate the expression of genes and the behavior of cells. DNA methylation can be epigenetically regulated by histone modification. For example, enhancer of zeste homolog 2 (EZH2), a histone methyltransferase, can promote DNA methylation by recruiting DNMTs in the target promoter region and then result in gene silencing [42]. Besides, lncRNAs regulate gene expression through interaction with histone modification enzymes, DNA methyltransferases [43, 44], or miRNA [45]. For example, some lncRNAs such as ANRIL are reported to regulate gene expression by recruiting the polycomb group of proteins, which can lead to heritable gene silencing through di- or trimethylation of lysine 27 of histone 3 (H3K27me2/3) [43, 44, 46]. In addition, lncRNAs can regulate the repression activity of miRNAs on mRNA [47]. Therefore, these regulatory mechanisms together constitute an epigenetic network, which regulates the expression of genes without changing the DNA sequence and affects the fate of cells.

## 3. DPSCs

### 3.1. Identification of DPSCs

DPSCs can be obtained from the dental pulp of permanent teeth extracted owing to impaction, orthodontic reason, or periodontitis. Similar to MSCs, DPSCs also express mesenchymal cell markers, like CD29, CD44, CD73, CD90, CD105, CD106, CD146, STRO-1, and aldehyde dehydrogenase 1 [48–52]. Meanwhile, DPSCs display negative or low expression of hematopoietic markers, CD14 or CD11b, CD19, CD34, CD45, and HLA-DR [50, 53, 54], which meets the minimal criteria for defining multipotent mesenchymal stromal cells proposed by the International Society for Cellular Therapy in 2006 [55]. However, DPSCs are a group of heterogeneous cells, and lots of the markers are not expressed in DPSCs consistently. Cells with different surface markers may have different characteristics. Therefore, purification of DPSCs is important for successful clinical application. Specific cell surface markers can facilitate the isolation of specific subsets of DPSCs, which can subsequently differentiate into specific cell types for clinical use. For example, single CD271^+^ DPSCs isolated by fluorescence-activated cell sorting have been found to have higher odontogenic potential [56].

### 3.2. Differentiation and Clinical Potential of DPSCs

**Figure 1:**
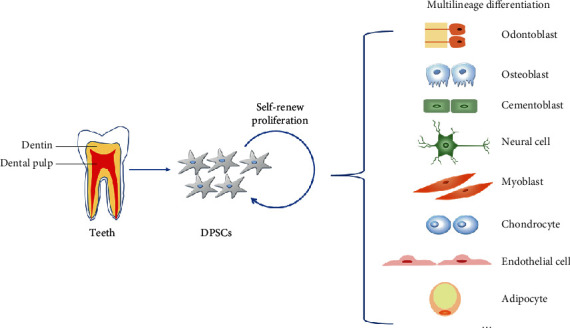
The multilineage differentiation potential of DPSCs. DPSCs can differentiate into odontoblasts, osteoblasts, cementoblasts, neural cells, myoblasts, chondrocytes, endothelial cells, adipocytes, etc.

The potential application in tissue engineering and regenerative medicine of MSCs has been widely proved. Over the past decades, bone marrow MSCs (BMMSCs), as a kind of MSCs, have become a focus of interest in regenerative medicine because of their multilineage differentiation ability. Recently, due to their easy accessibility, DPSCs have gradually come into the field of regenerative medicine. Compared to BMMSCs, DPSCs have better viability and higher capacity of odontogenic and neurogenic differentiation, but lower capacity to differentiate into chondrocytes [57–60]. When transplanted into immunocompromised mice, DPSCs can form dentin-like tissue, while BMMSCs form lamellar bone [61]. DPSCs can also differentiate into various kinds of cells, including osteoblasts, odontoblasts, adipocytes, endothelial cells, neurons, myocytes, and chondroblasts [9]. In addition, it has been proved that DPSCs can retain their properties even after two years of cryopreservation [62]. Therefore, more and more attention is paid to the differentiation and clinical potential of DPSCs in regenerative medicine (Figure 1).

Since DPSCs were first separated from human impacted third molars' pulp and cultured *in vitro* in 2000 [8], a series of studies demonstrated the self-renewal capability, multilineage differentiation potential, and clonogenic efficiency (colony-forming unit fibroblast) of DPSCs [8, 63, 64]. The osteo/odontogenic differentiation potential of DPSCs is the most widely reported. Both *in vitro* and *in vivo* experiments showed that DPSCs are able to differentiate into osteo/odontoblasts and form bone and dentin tissues [65–67]. When DPSCs are cultured in osteo/odontogenic induction medium, a group of proteins related to mineralization tissues are upregulated. Among them, dentin sialophosphoprotein (DSPP) and dentin matrix phosphoprotein 1 (DMP1) are considered to be specific markers of odontoblasts [68, 69]. While alkaline phosphatase (ALP), type I collagen (Col I), osteopontin (OPN), osteocalcin (OCN), and osterix (OSX) are associated with osteoblastic proliferation and differentiation [69]. As a result of origination from migrating neural crest cells, DPSCs can express some neural crest developmental genes and have the ability to differentiate into neural cells. When cultured in neuronal inductive conditions for an extended period of time, DPSCs exhibit a neuronal morphology and express neuronal-specific markers such as PSA-NCAM, *β*-III tubulin, neurofilament-M, and nestin, showing the ability to generate a sodium current consistent with functional neuronal cells [70]. Moreover, DPSCs transplanted in vitro can generate functional neurons and improve nerve regeneration [71, 72]. In addition, several studies also showed that DPSCs exhibit the capacity to acquire the phenotype of endothelial cells and generate vascular-like structures [73–76]. When cultured in a 3D fibrin mesh, DPSCs display endothelial cell-like features and form capillary-like structures [77]. After exposure to VEGF, endothelial-specific markers like Flt-l and KDR are increased, together with the occurrence of ICAM-l and the von Willebrand factor-positive cells [77]. After DPSCs have been cultured by using the “pellet culture” technique and chondrogenic medium, the structure of pellets is consistent with the structure of cartilage, and the Alcian blue staining of the extracellular matrix in the center of the pellets indicates the existence of highly sulfated glycosaminoglycans, demonstrating chondrogenic differentiation of DPSCs [9, 50, 78]. After being cultured in specific condition for several weeks, DPSCs elongate and display a myoblast-like phenotype. These DPSCs express specific myocytic immunohistochemical markers such as MyoD1, myosin, and MHC [79, 80]. Other than the myogenic potential, DPSCs also preserve the capability to differentiate into adipocytes [81, 82] and pancreatic cell lineage [83, 84].

Based on the multidirectional differentiation potential of DPSCs and their easy availability, the application of DPSCs in tissue engineering and diseases is increasingly being explored. DPSCs have been shown to form a dentin/pulp-like complex in immunocompromised mice [8]. Moreover, DPSCs in prevascularized, scaffold-free, microtissue spheroids can successfully regenerate vascular dental pulp-like tissue, which provides a new strategy for endodontic treatment and makes dentin-pulp regeneration possible [85]. The clinical application potential of DPSCs is not only in dentistry but also in treatments for other diseases, such as craniofacial bone defects [86], muscle regeneration [87], myocardial infarction [88], Alzheimer's disease [89], nervous system injuries [90], Parkinson's disease, diabetes [91], stress urinary incontinence [80], osteoarthritis [92], and liver diseases [93].

## 4. Epigenetic Mechanisms in DPSCs

Epigenetic regulation can influence the differentiation potential and proliferation of DPSCs. It is thus vital to understand the epigenetic mechanisms beneficial to the clinical application of DPSCs.

### 4.1. DNA Methylation

**Figure 2:**
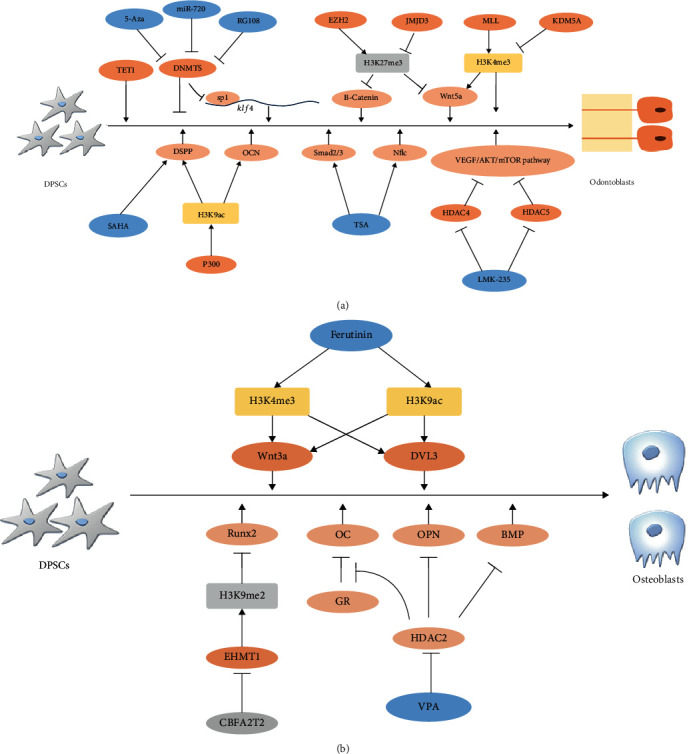
The modulation of DNA methylation and histone modifications during the odontogenic and osteogenic differentiation in DPSCs.

DNA methylation, one of the best-studied epigenetic modifications, is often related to gene silencing and regulation of stem cell fate. A series of studies also reported specific regulatory effects of DNA methylation in DPSCs (Table 1 and Figure 2).

Adult stem cells can be reprogrammed to induce pluripotent stem (iPS) cells and be applied to clinical therapy. During this process, DNA methylation plays a critical part [94]. In a genome-wide DNA methylation analysis, DPSCs exhibited DNA methylation profile closer to human embryonic stem (ES) cells and iPS cells [95]. Among these genes, overexpression of PAX9 and knockdown of HERV-FRD improved the efficacy of iPS generation from DPSCs. These results indicate the reprogramming potential of DPSCs into iPS and the role of epigenetic mechanisms in this process [96].

DPSCs, with multilineage differentiation potential, can differentiate into various kinds of cells under different environments. DNA methylation patterns may affect the process by regulating gene expression in DPSCs. The osteogenic genes with different DNA methylation statuses are associated with osteogenic differentiation potential [97]. Although DPSCs, DFSCs, and PDLSCs share almost similar DNA methylation patterns, some genes related to the development of the skeletal system, like SMAD3 and CD109, exhibit differential methylation profiles leading to variation in osteogenic capacities [97]. The differentiation potential of DPSCs varies with changes in the activity of DNMTs. Upon treatment with 5-Aza-2′-deoxycytidine (5-Aza-CdR), a DNA methyltransferase inhibitor, the proliferation capacity of DPSCs is suppressed. However, 5-Aza-CdR upregulates the odontogenic markers (DSPP and DMP1) and transcription factors (RUNX2, DLX5, and OSX), increases alkaline phosphatase (ALP) activity, and accelerates the formation of calcified nodules, which indicates an enhanced odontogenic differentiation potential [98]. Studies have shown that DNA methylation impacts the transactivation of transcription factor (TF) on its target gene. Inhibition of DNMTs causes demethylation of the *Klf4* promoter region, leading to enhanced binding of SP1, a transcriptional factor that upregulates the expression of *Klf4*. Krüppel like factor 4 (KLF4) has been proved to be vital for odontogenic differentiation [99]. Besides, the myogenic differentiation is also improved after treatment with 5-Aza-CdR [100]. Signs of muscle regeneration can be observed when DPSCs with the pretreatment of 5-Aza-CdR are applied to the muscle injury/regeneration model [101]. DNA demethylation enzymes can also affect the fate of DPSCs, especially TET1. TET1, existing in both the nucleus and the cytoplasm of the DPSCs, is a DNA dioxygenase and can promote DNA demethylation. The expression of TET1 increases during early cell passaging (<6^th^ passages) and then decreases. TET1 is also increased during odontogenic induction [102]. When TET1 is knocked down, the proliferation and odontogenic differentiation are suppressed [103]. Furthermore, TET1 can enhance odontogenic differentiation through the regulation of *FAM20C* demethylation and upregulation of the FAM20C expression [104].

Inflammation can occur in the dental pulp because of bacterial infection or trauma. This leads to the activation of a series of defense responses in DPSCs, including increased expression of inflammatory-related factors, odontogenic differentiation, and formation of restorative dentin. DNA methylation is involved in this process. During lipopolysaccharide- (LPS-) induced inflammation, DNMT1 mRNA and protein levels are reduced in DPSCs. DNMT1 can affect the MyD88 gene promoter methylation and downregulate the miR-146a-5p expression. Further research found that depletion of DNMT1 can enhance the inflammatory response through activation of the NF-*κ*B pathway [105, 106]. Similarly, the expression of proinflammatory cytokines, including GM-CSF, interleukin- (IL-) 6, RANTES, IL-8, and MCP-2, is upregulated, and the MAPK and NF-*κ*B signaling pathways are activated by 5-Aza-CdR in the LPS-treated DPSCs. Furthermore, 5-Aza-CdR decreases the levels of 5mc in the *TRAF6* promoter in DPSCs. These results indicate that 5-Aza-CdR accelerates the inflammatory process by the activation of TRAF6 [107]. Besides, TET2 improves the inflammatory response in the DPSCs by regulating the levels of 5hmC on the MyD88 promoter [108].

The above results indicate the critical role of DNA methylation in the differentiation and proliferation of DPSCs both *in vitro* and *in vivo*. However, further researches are necessary to explore the specific mechanism for the regulation of DPSCs through DNA methylation, so as to apply DPSCs safely and effectively in clinical treatment and tissue engineering.

### 4.2. Histone Modification

Histone modification often happens on the tail of histones and can turn genes on or off. Here, we conclude the regulation on DPSCs of histone modification (Table 2 and Figure 2).

#### 4.2.1. Histone Methylation

Histone methylation, mainly occurring in lysine or arginine residues located at the histone tails, is widely reported to modulate stem cell maintenance and differentiation. It has also been demonstrated in DPSCs. When comparing the epigenetic states between the DPSCs and the dental follicle cells (DFCs), H3K27me3-mediated repression of odontogenic-related genes, *DSPP* and *DMP1*, can be seen in the DFCs, but not in DPSCs. In accordance with the results, in osteoinductive conditions, DPSCs exhibit higher expression of both DSPP and DMP1, which indicates higher odontogenic ability [109]. EZH2, a histone methyltransferase, is responsible for repressive H3K27me3. During odontogenic differentiation, EZH2 decreases with the level of H3K27me3. Overexpression of EZH2 impairs the odontogenic differentiation; however, overexpression of EZH2 without methyltransferase activity does not affect the odontogenic differentiation of DPSCs. When *β*-tricalcium phosphate/DPSCs transfected with siEZH2 are transplanted under the skin of nude mice, the formation of mineralized tissue is improved. Further, the results of a CHIP assay suggested that EZH2 downregulates the expression of *β*-catenin by increasing the levels of H3K27me3 on the promoter region of *β-catenin*, eventually suppressing the Wnt/*β*-catenin signaling pathway that is critical for odontogenic differentiation [110]. EZH2 is also related to the proliferation, osteogenic differentiation, and inflammatory response of DPSCs. Under the appropriate inflammatory stimulation, DPSCs can differentiate into odontoblasts and migrate to the infected site to generate reparative dentin. In the infected cells, EZH2 and H3K27me3 are decreased. EZH2 inhibition can suppress IL-1b, IL-8, IL-6, and proliferation of DPSCs upon inflammatory irritation but enhances the osteogenic differentiation. These results prove that EZH2 inhibits osteogenic differentiation and enhances the inflammatory response and proliferation [111]. Another repressive histone methylation, H3K9, is also associated with osteogenic differentiation of DPSCs. The euchromatin histone methyltransferases-1 (EHMT1) can repress gene transcription and regulate cell differentiation through H3K9 dimethylation (H3K9me2) [112]. During the BMP-2-induced osteogenic differentiation, the level of H3K9me2 on the promoter of *Runx2* is downregulated by corepressor core-binding factor, runt domain, alpha subunit 2, translocated to, 2 (CBFA2T2). Knockdown of CBFA2T2 upregulates the expression of EHMT1 and increases the level of H3K9me2; however, the osteogenic differentiation is impaired [113]. The osteogenic differentiation of DPSCs is also regulated by the active mark trimethylation of lysine 4 of histone 3 (H3K4me3). Ferutinin, a daucane phytoestrogen, enhances the levels of H3K4me3 and H3K9ac on the promoters of *Wnt3a* and *DVL3* genes in DPSCs and improves the osteogenic differentiation by activating the Wnt/*β*-catenin signaling pathway [114]. These results demonstrated that histone modifications, such as H3K9me3, H3K27me3, and H3K4me3, are closely related to the differentiation process of DPSCs, especially the osteo/odontogenic differentiation.

Histone methylation is also a reversible process. There are various demethylases that can remove the methyl groups from histone. The Jumonji domain-containing protein D3 (JMJD3), also called lysine-specific demethylase 6B (KDM6B), can specifically demethylate H3K27me2/3 to regulate gene expression and modulate odontogenic differentiation through various mechanisms [115]. Overexpression of JMJD3 can enhance odontogenic differentiation, while the JMJD3 inhibition by alcohol impairs the odontogenic differentiation [116]. During the odontogenic-induction process, JMJD3 removes silencing H3K27me3 marks on the promoters of *BMP2*, and thus, the expression of transcription protein related to odontogenic differentiation BMP2 is activated [117]. The “bivalent domains,” containing both active mark H3K4me3 and repressive mark H3K27me3, are localized at the promoter regions of the *Wnt5a* gene. These modifications maintain the *Wnt5a* gene in a poised state, and under certain stimuli, the gene transcription is activated or repressed by the resolution of these marks [32, 118]. During odontogenic induction in DPSCs, the H3K27me3 on the *Wnt5a* promoter is removed by JMJD3, and Wnt5a is activated. The depletion of JMJD3 upregulates the level of H3K27me3, suppresses the expression of Wnt5a, and impairs the odontogenic differentiation. Besides, JMJD3 is important for H3K4me3 through the interaction with H3K4me3 methylases, mixed-lineage leukemia (MLL) complex [119]. Another HDMs, lysine-specific demethylase 5A (KDM5A), is specific for the active mark H3K4me3 [120]. The depletion of KDM5A can upregulate the level of H3K4me3 on the promoter of the odontogenic marker gene, including *DMP1*, *OSX*, *OCN*, and *DSPP*, and improve the odontogenic differentiation. These results indicate that H3K4me3 is also associated with odontogenic differentiation [121]. As mentioned above, HDMs can regulate gene expression and affect the fate of DPSCs through their specific demethylase activity.

#### 4.2.2. Histone Acetylation

Histone acetylation, which is usually considered to loosen the chromatin structure and facilitate gene transcription, profoundly impacts the differentiation and proliferation of different cells [122–124], including DPSCs. HAT improves the odontogenic differentiation of DPSCs by increasing the histone H3 acetylation of *DSPP* genes [125]. p300, a member of the lysine acetyltransferase 3 family, transfers the acetyl group to lysine residues. p300 upregulates the expression of SOX2 and NANOG in DPSCs, which is critical for maintaining the self-renewal and pluripotency of SCs through the enhancement of the transcriptional activities of the promoter. On the contrary, overexpression of p300 in DPSCs contributes to the reduction of odontogenetic markers, such as DSPP, OCN, DSP, OPN, and DMP1. However, when DPSCs are cultured in the odontoblastic induction medium, overexpression of p300 lacking the HAT domain increases the H3K9ac level on the promoter of *DSPP* and *OCN* and enhances the odontoblastic differentiation. Therefore, p300 is critical for the stemness of DPSCs by regulating the expression of SOX2 and NANOG and acts as a coactivator to upregulate the level of H3K9ac on the promoter of *DSPP* and *OCN* to promote odontogenic differentiation [126]. Consistent with the above results, another study found that when p300 is knocked down in DPSCs, proliferation and odontogenic differentiation are inhibited [127]. Besides, histone acetylation upregulated by photobiomodulation therapy can induce the proliferation of DPSCs [128].

In addition to HATs, histone acetylation levels are also affected by HDACs, which can transfer acetyl groups from histones, resulting in histone hypoacetylation and packed chromatin [123]. HDACs are classified into four categories, of which classes I (HDAC1,2,3,8), II (HDAC4,5,6,7,9,10), and IV (HDAC11) are zinc-dependent enzymes [129], while class III HDACs, often referred to as sirtuins, are nicotinamide adenine dinucleotide-dependent enzymes [130]. HDACs are also closely related to the differentiation of DPSCs. HDAC6 promotes the odontogenic differentiation of DPSCs, and when HDAC6 is knocked down, the odontogenic differentiation is impaired [131]. During the odontoblast differentiation, H3K9ac and H3K27ac are upregulated and p300 is increased, while HDAC3 is decreased [132]. These results indicate that odontoblast differentiation is coregulated by HATs and HDACs. To further explore, it is found that KLF4, a transcriptional factor, has a transactivation domain that binds directly to the target gene promoter and recruits coactivators like p300 or corepressors like HDAC3 [133]. The data of a CHIP analysis revealed that when dental pulp cells are induced into odontoblasts, HDAC3 mainly interacts with KLF4 on the promoter of *Dmp1* and *Sp7* on day 0 of induction, while p300 interacts on day 7 of induction. These results reveal that KLF4 can regulate the odontoblast differentiation by affecting the histone acetylation on the promoter regions of *DMP1* and *Sp7* and by interacting with p300 and HDAC3 [134].

HDAC inhibitors (HDACis) regulate gene expression by modulating the level of histone acetylation and have been widely used in cancer therapy [135]. HDACis can affect the differentiation and proliferation of DPSCs and may have potential applications in dental restoration [136]. Trichostatin A (TSA), a hydroxamic acid, inhibits the activity of all HDACs, except class IIa. TSA affects the proliferation of DPSCs in a dose-dependent manner and promotes the osteo/odontogenic differentiation through the upregulation of Smad2/3 and nuclear factor I-C- (Nfic-) related pathways. The proliferation of DPSCs is increased on exposure to 2 nmol/L and 20 nmol/L of TSA via the activation of the JNK/c-Jun pathway; however, higher concentrations of TSA lead to apoptosis. A 20 nmol/L solution of TSA can promote migration and adhesion of DPSCs [137–140]. Valproic acid (VPA), the short-chain fatty acid, can inhibit class I HDACs. Similar to TSA, the effect of VPA on DPSCs is dose-dependent, and at a certain concentration, it can improve the proliferation, adhesion, and migration of DPSCs [137]. In addition, VPA increases the mineralization and osteo/odontogenic differentiation [138, 139]. VPA increases the expression of OPN and BMP but decreases OCN, a late-stage marker of osteogenic differentiation, via HDAC2, which indicates that VPA promotes early differentiation of osteogenesis but does not promote terminal differentiation. In addition, VPA causes DPSCs to generate a well-organized bone tissue structure *in vivo*. Several studies have reported that glucocorticoid receptor (GR) is critical for this regulation. HDAC2 binds to GR and inhibits its translocation into the nucleus, but when HDAC2 is inhibited by VPA, GR can enter the nucleus and thus affect the expression of the OC [141, 142]. Suberoylanilide hydroxamic acid (SAHA), a pan inhibitor of HDACs, increases the expression of DSPP via the activation of Nfic and enhances odontogenic differentiation in DPSCs [143]. LMK-235, a specific inhibitor to HDAC4 and HDAC5, improves odontogenic differentiation through the VEGF/AKT/mTOR pathway without affecting the proliferation of DPSCs [144].

Histone acetylation regulates various physiological processes of DPSCs and affects their fate. HDACis may have potential applications in the treatment of mineralized regeneration; however, further research is needed in this context.

### 4.3. ncRNAs

#### 4.3.1. miRNAs

miRNAs specifically recognize the target mRNA through base complementation and affect its stability by binding to the 3′UTR, which eventually leads to suppression of protein translation. miRNAs are related to stemness, cell reprogramming, and differentiation of various cells, including DPSCs (Table 3).

miRNAs are vital in regulating the proliferative capacity of DPSCs. Foxq1, a transcriptional factor, regulates cell cycle and promotes the stemness and proliferation. When the proliferation of DPSCs is promoted by calcium hydroxide, the expression of Foxq1 is also increased; however, miR-320b, which negatively regulates Foxq1, is decreased. Therefore, miR-320b can mediate the proliferation of DPSCs via Foxq1 [145]. The transcriptional coactivator with PDZ-binding motif (TAZ) is reported to be essential for the proliferation of DPSCs. TAZ can be silenced by miR-584, which binds directly to *TAZ* mRNA and in turn suppresses the proliferation [146].

The senescence of DPSCs is accompanied by a decline in proliferation and differentiation ability, affecting the clinical use of DPSCs. miR-152 is upregulated with the senescence of DPSCs. miR-152 targets sirtuin 7 (SIRT7), which modulates gene expression by regulating histone deacetylase activity, and induces DPSC senescence [147]. Apoptosis is a genetically programmed cell death. miR-224-5p protects DPSCs from apoptosis by silencing Rac family small GTPase 1 (Rac1), which has been proved to induce apoptosis [148]. Besides, miR-224-5p can improve the migration and proliferation of DPSCs [149].

The miR-143 family negatively modulates the odontogenic and osteogenic differentiation of DPSCs. miR-143-5p impairs the odontogenic differentiation by targeting RUNX2 via the OPG/RANKL signaling pathway [150]. It has been reported that miR-143-5p binds to MAPK14 and reduces its expression. Thus, miR-143-5p knockdown increases MAPK14 expression and activates the p38 MAPK signaling pathway, consequently enhancing the odontogenic differentiation [151]. In addition, by directly targeting tumor necrosis factor-*α* (TNF-*α*), miR-143 blockades the NF-*κ*B signaling pathway and suppresses the osteogenic differentiation [152]. Some other miRNAs are also associated with the osteo/odontogenic differentiation of DPSCs. miR-140-5p is decreased when DPSCs are induced into odontoblasts, and miR-140-5p mimic can impair the odontogenic differentiation through suppressing the Wnt1/*β*-catenin signaling pathway by targeting Wnt1 [153]. Insulin-like growth factor 1 has been proved to induce the proliferation and osteo/odontogenic differentiation of DPSCs via activation of the P38 MAPK and JNK pathways. However, overexpression of let-7c can reverse the process but not affect the proliferation by suppressing the insulin-like factor 1 receptor (IGF-1R). During this process, the JNK/P38 MAPK pathway is also repressed [154]. Besides, miR-215 and miR-219a-1-3p negatively modulate the osteogenic differentiation of DPSCs through downregulation of heat shock protein B8 (HspB8) [155]. In an osteoinductive environment, miR-218, which suppresses the osteogenic differentiation of DPSCs by targeting *RUNX2*, is decreased [156]. miR-218 also suppresses the odontogenic differentiation through the MAPK/ERK pathway. On delivering miR-218 inhibitor into DPSCs by a newly designed magnetic nanocarrier, GCC-Fe_3_O_4_, mineralization nodules are increased, which is a novel application of DPSCs [157]. Compared to the undifferentiated DPSCs, miR-720 is highly expressed in differentiated DPSCs. Further, it has been found that miR-720 decreases the proliferation and enhances the odontogenic differentiation of DPSCs through directly repressing NANOG and indirectly silencing NANOG by induction of DNMT3A and DNMT3B [158]. In addition, during odontogenesis of DPSCs, miR-508-5p is gradually decreased, while glycoprotein nonmetastatic melanoma protein B (GPNMB), also called osteoactivin, is increased. Further research demonstrated that knockdown of miR-508-5p can promote odontogenesis in DPSCs via upregulation of GPNMB [159]. In conclusion, miRNAs affect the osteo/odontogenic differentiation of DPSCs by regulating various key molecules in the osteo/odontogenesis process.

Growing evidence indicates that miRNAs play a critical role in angiogenic processes [160]. In particular, miR-424 is expressed in a sequential manner during the endothelial differentiation of DPSCs. Overexpression of miR-424 inhibits endothelial differentiation. Thus, miR-424 negatively regulates the endothelial differentiation of DPSCs [161].

Interestingly, the expression of miR-143 and miR-135 is significantly downregulated in myoblast DPSCs induced by 5-Aza. The addition of miR-143 or miR-135 inhibitors to culture medium stimulates the myocytic properties of DPSCs, which eventually fuse to form myotube [162]. Additionally, miR-139-5p regulates the skeletal myogenic differentiation of human DPSCs by interacting with the Wnt/*β*-catenin signaling pathway [79]. These outcomes reveal that miRNAs are essential for the induction of myogenic differentiation of DPSCs.

The inflammatory microenvironment can interact with DPSCs and affect the fate of DPSCs [163, 164]. A series of studies reported that miRNAs are involved in the interaction between inflammatory microenvironment and DPSCs. By comparing the expression of miRNAs between healthy and inflamed pulp, 79 differentially expressed miRNAs have been identified. Among them, miR-223-3p is significantly upregulated. Furthermore, overexpression of miR-223-3p increases DSPP and DMP1 but suppresses Smad3. According to the dual-luciferase assay, miR-223-3p promotes odontogenic differentiation by targeting Smad3 and enhances pulpal healing [165]. LPS, a major pathogenic factor of Gram-negative bacteria, is closely related to pulpitis caused by caries. In the DPSCs treated by LPS, the proinflammatory cytokines, such as TNF-*α* and IL-6, are increased, the viability is decreased, and osteo/odontogenic differentiation is impaired. In the LPS-treated DPSCs, the expression of miR-506 is upregulated, and TLR-4 pathway is activated. However, miR-506 knockdown attenuates the inflammatory response and suppresses the TLR-4 pathway by upregulating SIRT1 [166]. These inflammatory responses are reversed in the presence of let-7c-5p overexpression in LPS-induced DPSCs. It has been reported that let-7c-5p protects DPSCs from inflammation by directly repressing DMP1 and promotes the osteogenic differentiation through inhibition of HMGA2/PI3K/Akt signaling [167, 168]. Besides, DPSCs treated with TNF-*α* exhibit increased expression of Fyn, a member of the protein tyrosine kinase Src family, which is related to inflammation and odontogenesis; however, the expression of miR-125a-3p is decreased. It has been found that miR-125-3p can reverse the inflammatory response and enhance odontogenic differentiation by repressing Fyn [169]. Moreover, a certain concentration of LPS can improve the proliferation, adhesion, and migration of DPSCs and differentiation of odontoblast through Toll-like receptor (TLR-4), ERK, and P38 MAPK signaling pathways [164, 170]. During the LPS-induced odontoblastic differentiation, the expression of miR-140-5p is downregulated. When miR-140-5p is overexpressed, the differentiation and proliferation of DPSCs are impaired. A luciferase reporter analysis demonstrated that miR-140-5p can bind to the 3′UTRs of the *TLR-4* mRNA, and the inhibition of TLR-4 can reverse the impact on the proliferation and differentiation of DPSCs via inhibition of miR-140-5p. These outcomes indicate that miR-140-5p impairs the differentiation and proliferation of DPSCs induced by LPS [171]. The cytokine TNF-*α* enhances odontogenic differentiation at low concentrations (1-10 ng/mL) and suppresses the same at high concentrations (50-100 ng/mL). Consistent with the above results, increased expression of miR-21, as well as signal transducer and activator of transcription 3 (STAT3), is observed at low concentrations of TNF-*α*, while the opposite results are observed at high concentrations. It is noteworthy that miR-21 and STAT3 form a positive feedback loop to regulate odontogenic differentiation [172]. These results reveal that miRNAs are associated with the inflammatory response of DPSCs and also provide a new perspective for the treatment of pulpitis.

#### 4.3.2. lncRNAs

lncRNAs modulate gene expression at different levels and regulate the fate of DPSCs (Table 3).

As the donor's age increases, DPSCs are also gradually senescent. During this process, 389 lncRNAs are downregulated and 172 lncRNAs are upregulated, which also indicates the important role of lncRNAs in the senescence of DPSCs [173]. The lncRNA, antidifferentiation noncoding RNA (ANCR), also called differentiation antagonizing nonprotein coding RNA (DANCR), was first identified in 2012. It suppresses the differentiation and enforces the undifferentiation state of somatic progenitor populations [174]. Similarly, the inhibition of ANCR promotes the osteogenic, neurogenic, and adipogenic differentiation of DPSCs, without affecting the proliferation [175]. It has been reported that ANCR impairs the odontogenic differentiation of DPSCs by repressing of the Wnt/*β*-catenin signal pathway [176].

lncRNAs are also closely associated with odontogenic ability of stem cells. Through RNA-sequencing analysis, 108 lncRNAs are found to be downregulated and 36 lncRNAs are found to be upregulated in association with the loss of odontogenic differentiation potential [177]. In another research, when DPSCs are induced to differentiate into odontoblasts, the expression of 114 miRNAs and 132 lncRNAs is found to be altered. Through bioinformatics analyses, two lncRNA-associated ceRNA networks centered two odontogenic-related proteins, rhodopsin and Fibrillin 1 (FBN1), are found to be involved in the odontogenic differentiation of DPSCs. Further research reported that lncRNA G043225 improves the odontogenic differentiation by competitively inhibiting the repression activity of miR-588 on FBN1 as an endogenous miRNA sponge [178]. Besides, lncRNA H19 can upregulate S-adenosylhomocysteine (SAH), which is an inhibitor of S-adenosylmethionine-dependent methyltransferase, and downregulate DNA methylation levels [179]. Therefore, H19 upregulates the expression of distal-less homeobox3 (DLX3) and enhances the odontogenic differentiation of DPSCs through the downregulation of the methylation level on *DLX3* gene [180].

DPSCs are able to differentiate into osteoblasts and form bone tissue, which can be used in bone regeneration therapy. Studies have confirmed that lncRNAs are also associated with the osteogenic differentiation of DPSCs. Examination of the expression of lncRNAs during TNF-*α* induced osteogenic differentiation revealed 58 upregulated and 19 downregulated lncRNAs on day 7 and 73 upregulated and 60 downregulated lncRNAs on day 14 [181]. lncRNA colon cancer-associated transcript 1 (CCAT1) is initially discovered to participate in metabolic, migratory, and proliferative processes in some cancers [182]. Later on, it was found that CCAT1 can promote the proliferation and odontogenic differentiation of DPSCs. A luciferase assay suggested that CCAT1 directly bind to the miR-218 and negatively regulate the expression of miR-218 [183].

The angiogenic differentiation of DPSCs is important for pulp regeneration. During the angiogenic induction in DPSCs, 376 lncRNAs are significantly upregulated, including SMILR, while 426 lncRNAs are downregulated. These results indicate the critical role of lncRNAs in angiogenic differentiation [184]. Still, further research is warranted to explore specific mechanisms.

## 5. Conclusion

DPSCs have gained increased attention in the field of regenerative medicine owing to their multilineage differentiation potential and easy accessibility. In this review, we summarize the regulation of epigenetic modifications mainly including DNA methylation, histone modification, and ncRNAs in the differentiation and proliferation of DPSCs. While most studies were mainly conducted *in vitro*, further investigations, including *in vivo* experiments and animal disease models, would be needed to explore the clinical potential in disease treatment and regenerative medicine such as HDACis and DNMT inhibitors related to epigenetic modifications. In addition, some other epigenetic modifications such as RNA modification and chaperones have been shown to be involved in embryo development, cell differentiation, and pluripotency maintenance [185–188]. It would be necessary to reach deeper insights into the role of these epigenetic modifications in the modulation of DPSC fate.

## Figures and Tables

**Table 1:** DNA methylation in DPSCs.

Epigenetic modifier	Epigenetic mark	Results
TET1	DNA demethylation	TET1, existing both in the cytoplasm and nuclei of the DPSCs, can improve the proliferation and odontogenic differentiation [102–104].
5-Aza-CdR	DNMT inhibitor	The inhibition of DNA methylation by 5-Aza negatively regulates the proliferation and enhances the myogenic and odontogenic differentiation [98, 100, 101].
RG108	DNMT inhibitor	SP1 can improve the expression of KLF4 through binding to the demethylated promoter region during the odontoblastic differentiation [99].

**Table 2:** Histone modifications in DPSCs.

Epigenetic modifiers	Epigenetic marks	Targets	Differentiation
Histone methylation			
EZH2	H3K27me3	Wnt/*β*-catenin pathway	Inflammation, proliferation, osteogenic [110, 111]
EHMT1	H3K9me2	RUNX2	Osteogenic [113]
MLL	H3K4me3	Wnt5a	Odontogenic [119]
Histone demethylation			
KDM5A	H3K4me3	DMP1, DSPP, OSX, and OCN	Odontogenic [121]
KDM6B	H3K27me3	Wnt5a, BMP2	Osteo/odontogenic [116, 117, 119]
Histone acetylation			
HAT	H3 acetylation	DSPP	Odontogenic [125]
p300	H3K9ac	NANOG, SOX2, DSPP, OCN, Dmp1, and Sp7	Pluripotency, proliferation, odontogenic [126, 127, 132, 134]
Histone deacetylation			
HDAC3	H3K27ac	Dmp1, Sp7	Odontogenic [132, 134]
HDAC6			Odontogenic [131]

**Table 3:** ncRNAs in DPSCs.

ncRNAs	Targets	Differentiation
lncRNAs		
ANCR	p-GSK-3*β* and *β*-catenin	Odontogenic [175, 176]
H19	SAHH	Odontogenic [179, 180]
CCAT1	miR-218	Proliferation, osteogenic [183]
G043225	miR-588	Odontogenic [178]
miRNAs		
miR-224	Rac1	Migration, proliferation, apoptosis [148, 149]
miR-152	SIRT7	Senescence [147]
miR-140-5p	TLR-4, Wnt1	Proliferation, odontogenic [153, 171]
miR-720	NANOG	Proliferation, odontogenic [158]
miR-584	TAZ	Proliferation [146]
miR-320b	Foxq1	Proliferation [145]
miR-21	STAT3	Odontogenic [172]
miR-143, miR-143-5p	Runx2, MAPK14, TNF-*α*	Osteo/odontogenic, myogenic [150–152, 162]
miR-508-5p	GPNMB	Odontogenic [159]
miR-223-3p	Smad3	Odontogenic [165]
miR-506	SIRT1	Inflammation [166]
miR-218	RUNX2	Osteo/odontogenic [156, 157]
miR-215, miR-219a-1-3p	HspB8	Osteogenic differentiation [155]
let-7c, let-7c-5p	IGF-1R, DMP1	Osteo/odontogenic, inflammation [154, 167, 168]
miR-125-3p	Fyn	Odontogenic, inflammation [169]
miR-424	VEGF, KDR	Angiogenic [161]
miR-135		Myogenic [162]
miR-139-5p	Wnt/*β*-catenin signaling pathway	Myogenic [79]
